# Retrievable Scaffold Therapy Combined with Sirolimus-coated Balloon Angioplasty for Infrapopliteal Artery Disease: Final Results from the DEEPER LIMUS Trial

**DOI:** 10.1007/s00270-025-03987-y

**Published:** 2025-02-21

**Authors:** Leyla Schweiger, Katharina Gütl, Peter Rief, Clemens Reiter, Michael Janisch, Ido Weinberg, Raghu Kolluri, Larry E. Miller, Marianne Brodmann

**Affiliations:** 1 Division of Angiology, Department of Internal Medicine, Medical University of Graz, Graz, Austria; 2 Division of Neuroradiology, Vascular and Interventional Radiology, Department of Radiology, Medical University of Graz, Graz, Austria; 3 Vascular Medicine Section, Massachusetts General Hospital, Boston, MA USA; 4 Syntropic Core Lab, Columbus, OH USA; 5 Department of Biostatistics, Miller Scientific, 3101 Browns Mill Road, Ste. 6, #311, Johnson City, TN 37604 USA

**Keywords:** Drug-coated balloon, Infrapopliteal, Retrievable scaffold therapy, Sirolimus

## Abstract

**Purpose:**

To evaluate the safety and efficacy of retrievable scaffold therapy combined with sirolimus-coated balloon angioplasty for treating infrapopliteal artery lesions.

**Methods:**

The DEEPER LIMUS study enrolled Rutherford class 3 to 5 patients with infrapopliteal artery disease. Patients underwent vessel preparation with retrievable scaffold therapy followed by sirolimus-coated balloon angioplasty. The primary safety outcome measure was a composite of all-cause mortality, major amputation, or clinically driven target lesion revascularization at 6 months. Secondary outcome measures included acute vessel recoil, perioperative death, angiographic late lumen loss between post-procedure and 6 months, as well as primary patency, major amputation, change in Rutherford class, and wound healing through 12 months.

**Results:**

The study included 26 patients (mean age 71 years, 62% male, 88% Rutherford 5) with 28 treated infrapopliteal lesions (36% occluded, 54% TASC B/C). Acute vessel recoil, measured in seven patients, was negligible (2.4 mm after treatment vs. 2.3 mm 15 minutes after deployment). A 6-month primary safety event occurred in 11.5% of patients (1 all-cause mortality, 1 major amputation, 1 clinically driven target lesion revascularization). Late lumen loss at 6 months was 0.7±0.7 mm. At 12 months, primary patency was 89.5%, improvement in Rutherford class was observed in 68.2% of patients, 90% of patients were deemed very low/low major limb amputation risk based on Wound, Ischemia, and foot Infection (WIfI) scores, and no additional major amputations or clinically driven target lesion revascularizations were reported.

**Conclusions:**

Retrievable scaffold therapy combined with sirolimus-coated balloon angioplasty demonstrated promising safety and efficacy through 12 months in patients with infrapopliteal artery disease.

**Level of Evidence IV:**

Case Series.

**Graphical Abstract:**

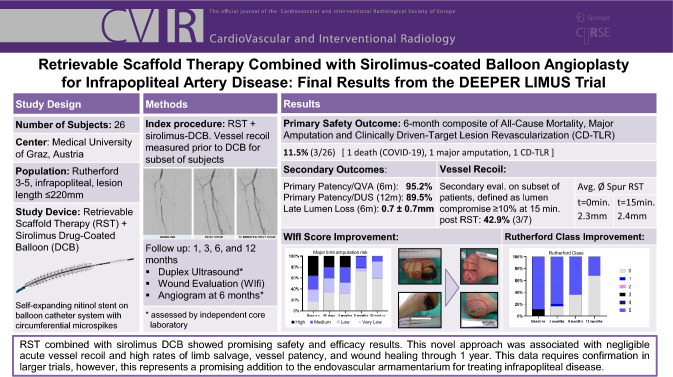

## Introduction

Peripheral artery disease affects over 200 million individuals worldwide [1], with chronic limb-threatening ischemia (CLTI) representing its most severe manifestation. Patients with CLTI typically present with rest pain, non-healing wounds, or gangrene due to advanced ischemia and often require revascularization of the infrapopliteal arteries to prevent amputation [2]. However, the management of infrapopliteal artery disease presents unique therapeutic challenges attributable to heavy plaque burden, severe calcification, diffuse lesions, and poor distal runoff. Thus, there remains a clinical need for new therapeutic approaches to improve outcomes in affected individuals.

In recent years, endovascular therapies for infrapopliteal artery revascularization have evolved to include a spectrum of options including percutaneous transluminal angioplasty (PTA), bare metal stents, drug-coated balloons (DCBs), and drug-eluting stents, among others. Given the limited high-quality evidence for these treatment options, PTA remains the primary approach for infrapopliteal artery revascularization. However, PTA is associated with suboptimal treatment durability [3, 4]. Moreover, findings from studies comparing these various interventional approaches to PTA have been inconsistent [5–8], indicating that no single approach has consistently demonstrated superior outcomes in this challenging patient population.

Yet drug-coated technologies continue to attract significant interest in the treatment of infrapopliteal artery disease, with ongoing efforts to improve localized drug delivery and transfer to the artery wall. The rationale behind their use lies in the localized delivery of antiproliferative agents to inhibit neointimal hyperplasia, which is a primary mechanism of restenosis following endovascular interventions. Recent concerns regarding the long-term safety of paclitaxel-coated devices in the peripheral vasculature [9, 10] have prompted interest in alternative drug coatings. Limus-derived drugs such as sirolimus and everolimus exert their effects through inhibition of the mammalian target of rapamycin, leading to cell cycle arrest and reduced smooth muscle cell proliferation [11]. However, effective local arterial delivery of limus drugs has historically proven challenging in the absence of a stent platform to facilitate vessel wall transfer [12]. To address this limitation, retrievable scaffold therapy (RST) incorporating a spur stent was developed to provide transient mechanical scaffolding, prevent vessel recoil, and facilitate drug transfer from DCBs to the artery wall, while avoiding a permanent metallic implant. The DEEPER LIMUS study was designed to evaluate the safety and performance of this system when combined with sirolimus-coated balloon angioplasty for treating patients with infrapopliteal artery disease.

## Methods

DEEPER LIMUS was a prospective, single-center, single-arm trial conducted at Medical University of Graz (Graz, Austria). The study population included adult patients presenting with Rutherford class 3 to 5 and *de novo* or restenotic infrapopliteal artery lesions. Eligible lesions were no more than 220 mm in length within reference vessels between 2.0 and 4.5 mm in diameter. Key exclusion criteria were planned target limb major amputation, osteomyelitis proximal to the phalanges, severe calcification precluding balloon angioplasty using the Peripheral Arterial Calcium Scoring System (PACSS), inflow lesion stenosis ≥50% unless successfully treated (residual stenosis ≤50%) prior to target lesion treatment, and angiographic evidence of thrombus within the target limb. The local ethics committee approved the study protocol, and all patients provided written informed consent. The trial was registered at https://www.ClinicalTrials.gov (NCT04162418) prior to patient enrollment.

**Fig. 1:**
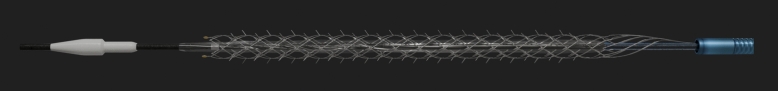
Retrievable Scaffold Therapy. This temporary intra-arterial scaffold consists of a balloon-expandable nitinol stent with circumferential microspikes that extend radially upon deployment, creating microchannels in the vessel wall to enhance antiproliferative drug uptake and retention from subsequent drug-coated balloon angioplasty

All patients were treated with RST followed by sirolimus balloon angioplasty. The RST used in this trial consisted of a self-expanding nitinol stent that is attached to a balloon catheter shaft, collapsed on the balloon within the 5.6-Fr delivery outer shaft, and delivered through a 6-F access sheath (Spur Peripheral Retrievable Scaffold System; Reflow Medical, Inc.; San Clemente, CA, United States) (Fig. 1). The device can be delivered via an ipsilateral or contralateral approach. The scaffold was available in 3 × 60 mm and 4 × 54 mm sizes. A key feature of the device is the circumferential microspikes extending from the outer surface of the stent. In preclinical models, RST was shown to create microchannels in the vessel wall, facilitate drug transfer into the deeper layers of the artery wall, inhibit neointimal proliferation, and reduce vessel recoil through controlled dilation of the diseased arterial wall [13].

After obtaining vascular access, patients underwent intravascular ultrasound, optical coherence tomography, or angiography to confirm eligibility including determination of reference vessel diameter and device sizing. Next, the target lesion was predilated with a standard commercially available PTA catheter. Following successful predilation, the scaffold was then advanced to the target lesion and deployed at nominal pressure (6 atm) for at least 2 minutes. The scaffold was then recaptured into the delivery system and removed from the vasculature. A commercially available sirolimus-based DCB (Magic Touch, Concept Medical, Tampa, FL, United States) sized 1:1 in diameter was then delivered and inflated for 2 minutes at the target lesion. Acute vessel recoil was measured 15 minutes after RST deployment in a subset of patients. This measurement was added as a study outcome through a protocol revision during enrollment. Thus, the analysis of acute vessel recoil involves the final 7 consecutive subjects enrolled after this revision.

Following the procedure, patients were prescribed dual antiplatelet therapy for a minimum of 6 months. Patients returned for clinical follow-up visits at 1, 3, 6, and 12 months. Duplex ultrasound was performed at each visit to assess vessel patency, and images were assessed by an independent core laboratory (VasCore, Boston, MA, United States). Angiography was additionally performed at the 6-month visit, with procedural and 6-month images assessed by an independent core laboratory (Syntropic Core Lab, Columbus, OH, United States) to measure late lumen loss (LLL), defined as the change from the post-procedural luminal diameter.

The primary outcome measure of the trial was a composite of all-cause mortality, major (above ankle) amputation, and clinically driven target lesion revascularization (CD-TLR) through 6 months. CD-TLR was defined as any reintervention at the target lesion associated with worsening Rutherford class or wound status. Secondary safety outcome measures included: a) freedom from perioperative death or target limb major amputation at 30 days and b) freedom from target limb major amputation at 6 and 12 months. Secondary efficacy outcome measures included: a) acute vessel recoil 15 minutes after RST measured with angiography in a subset of patients, b) LLL measured with angiography at 6 months, c) primary patency (lesion flow without CD-TLR) at 6 months by angiography, d) change in Rutherford score at 3, 6, and 12 months, and e) wound healing using the Wound, Ischemia, and foot Infection (WIfI) classification system, with determination of risk for major limb amputation [14]. Device success was defined as the successful delivery and retrieval of the RST device. Procedure success was defined as device success with freedom from an in-hospital major adverse event.

Categorical variables were reported as counts and frequencies, and continuous variables were reported as means and standard deviations. Statistical analyses were performed by independent biostatisticians using SAS version 9.4 (SAS Institute, Cary, NC, United States).

## Results

Between May 2020 and April 2022, 26 patients (mean age 71±9 years, 62% male) with infrapopliteal artery disease were enrolled in the trial. The most common comorbidities were hypertension (89%), diabetes (77%), and hyperlipidemia (77%). Three (12%) patients presented with Rutherford class 3, while 23 (88%) patients were classified as Rutherford class 5. A total of 28 infrapopliteal lesions were treated, with a mean lesion length of 61 ± 42 mm (range 30–170 mm). Of these, 36% were occluded, and 54% were classified as TASC B or greater (Table 1).

**Table 1:** Patient and procedural characteristics

Characteristic	Value*
*Demographics*	
Age, yrs	71 ± 9
Male sex	61.5% (16/26)
*Medical history*	
Hypertension	88.5% (23/26)
Hyperlipidemia	76.9% (20/26)
Diabetes mellitus	76.9% (20/26)
Tobacco use	61.5% (16/26)
Coronary artery disease	30.8% (8/26)
Chronic kidney disease	26.9% (7/26)
Osteomyelitis of index foot	19.2% (5/26)
Cerebrovascular disease	15.4% (4/26)
Myocardial infarction	15.4% (4/26)
Congestive heart failure	3.8% (1/26)
Planned amputation of index limb/toes	11.5% (3/26)
Previous amputation of index limb/toes	7.7% (2/26)
*Rutherford-Becker class*	
3	11.5% (3/26)
4	0.0% (10/26)
5	88.5% (23/26)
***Lesion characteristics***	
*TASC classification*	
A	46.4% (13/28)
B	35.7% (10/28)
C	17.9% (5/28)
*PACSS calcium score*	
0	75.0% (21/28)
1	17.9% (5/28)
2	7.1% (2/28)
Occlusion	35.7% (10/28)
Lesion length, mm	61 ± 42
*Treated artery*	
Anterior tibial	39.3% (11/28)
Tibioperoneal trunk	28.6% (8/28)
Posterior tibial	17.9% (5/28)
Peroneal	14.3% (4/28)
*Procedures*	
Procedure duration, min	67 ± 14
Fluoroscopy time, min	9 ± 6
Contrast volume, ml	72 ± 21
Spur stent treated length, mm	98 ± 40

^*^Values are mean ± SD or *n* (%).

DCB, drug-coated balloon; PASCC, Peripheral Arterial Calcium Scoring System; TASC, Trans-Atlantic Inter-Society Consensus.

Lesion crossing and scaffold delivery, deployment, and recapture were successful in all cases, and no in-hospital major adverse events occurred. Thus, device and procedural success rates were both 100%. In the subset of patients evaluated for acute vessel recoil (*n* = 7), recoil was negligible (2.4±0.2 mm after RST vs. 2.3±0.2 mm 15 minutes after deployment).

No patients were lost to follow-up during the trial. No device-related adverse events were reported during the study. A 6-month primary safety event occurred in 3 (11.5%) patients, including all-cause mortality (*n* = 1; 3.8%), major amputation (*n* = 1; 3.8%), and CD-TLR (*n* = 1; 3.8%). All-cause mortality was 0% during the 30-day perioperative period and 15.4% through 12 months. No additional major amputations or CD-TLRs occurred through 12 months.

**Fig. 2:**
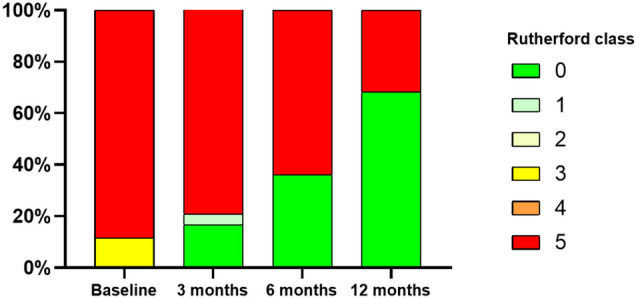
Change in Rutherford Classification over 12 Months after Treatment with Retrievable Scaffold Therapy in Combination with Sirolimus-coated Balloon Angioplasty. At baseline, 88.5% of patients were Rutherford class 5 and 11.5% were Rutherford class 3. At 12 months, 31.8% of patients were Rutherford class 5 and 68.2% were asymptomatic (Rutherford class 0)

**Fig. 3:**
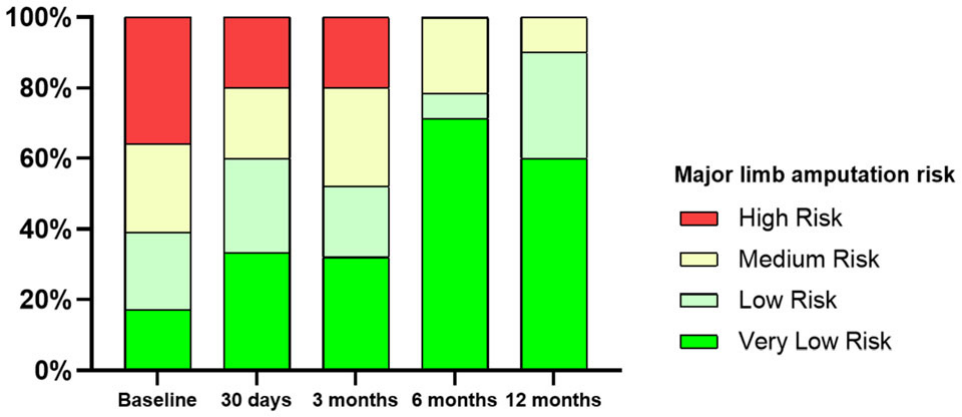
Change in Wound, Ischemia, and foot Infection (WIfI) score over 12 Months after Treatment with Retrievable Scaffold Therapy in Combination with Sirolimus-coated Balloon Angioplasty. At baseline, 60.7% of patients were deemed medium or high risk. At 12 months, 10.0% of patients were deemed medium risk and none were high risk

The mean LLL at 6 months, measured by minimal lumen diameter, was 0.7±0.7 mm, corresponding to a mean percentage change of 31±30%. Primary patency by angiography at 6 months was 95.2%. By duplex ultrasound, primary patency was 91.3% at 6 months and 89.5% at 12 months. Improvement in Rutherford class was observed in 68.2% of patients at 12 months, with no patients demonstrating worsening (Fig. 2). Among Rutherford 5 patients, the mean WIfI wound scores were 1.8±0.8 at baseline, 1.0±1.1 at 6 months, and 0.8±0.9 at 12 months, and the mean WIfI total scores were 2.8±1.1, 1.5±0.8, and 1.5±0.7, respectively. At 12 months, 10% of patients were deemed at medium risk and none at high risk for major limb amputation based on WIfI score classification (Fig. 3).

## Discussion

The DEEPER LIMUS trial introduces a novel approach to treating infrapopliteal artery disease by combining RST with sirolimus-coated balloon angioplasty. This strategy addresses several key challenges in the management of infrapopliteal disease, including the need for optimal lesion preparation, effective drug delivery, reduction of vessel recoil, and the avoidance of permanent implants. The key findings from this trial included negligible acute vessel recoil, low rates of major amputation and CD-TLR, sustained patency, and significant improvements in Rutherford class and wound healing over 1 year. The promising safety and efficacy outcomes observed in this study suggest that this approach may be a useful addition to the endovascular treatment options for infrapopliteal artery disease.

Vessel recoil remains a significant limitation of endovascular treatment of infrapopliteal arteries and is associated with acute luminal loss, treatment failure, restenosis, and the need for reintervention [15–18]. The negligible acute vessel recoil observed after RST deployment is a key finding of this study and represents an improvement over the 97% recoil previously reported with PTA alone [15]. These promising elastic recoil results likely contributed to the favorable patency outcomes observed. The primary patency rate of 89.5% at 12 months exceeds rates with PTA [4, 8, 19–21] or DCB [20–22] alone (Table 2). This potential benefit may be due to the combined beneficial effects of temporary scaffolding and enhanced localized drug delivery. The retrievable scaffold provides acute luminal gain and vessel support, while the radial microstructures create microchannels in the arterial wall. Subsequent application of sirolimus-coated balloon angioplasty leverages this modified vessel architecture, potentially enhancing drug uptake and retention [13]. The ability of RST to minimize vessel recoil and facilitate drug transfer into the artery wall while avoiding the long-term risks associated with permanent stent implantation represents a novel, multifaceted approach to managing complex infrapopliteal disease.

**Table 2:** Primary patency with retrievable scaffold therapy in combination with sirolimus-coated balloon angioplasty compared to meta-analysis-derived estimates with DCB and PTA in Infrapopliteal arteries

Study	6 months	1 year
*RST + sirolimus DCB*		
Current study	91%	90%
*DCB*		
Guo [2022] [20]	68%	74%
Giannopoulos [2020] [22]	†	64%
Ipema [2020] [21]	†	67% *
*PTA*		
Snyder [2023] [19]	68%	66%
Guo [2022] [20]	35%	27%
Nugteren [2022] [8]	†	59%
Ipema [2020] [21]	†	38% *
Romiti [2008] [4]	65%	58%

^*^Values are restenosis rates; primary patency was not reported.

^†^Values not reported.

DCB, drug-coated balloon; PTA, percutaneous transluminal angioplasty; RST, retrievable scaffold therapy.

The choice of sirolimus as the anti-restenotic agent in this study warrants additional discussion. While paclitaxel-based devices have demonstrated efficacy in peripheral interventions, safety concerns [9, 10] have prompted interest in alternative antiproliferative agents. Sirolimus offers potential advantages over paclitaxel due to its different mechanism of action. Unlike paclitaxel, which acts as a microtubule stabilizer and disrupts cell division [23], sirolimus inhibits the mammalian target of rapamycin pathway [11]. By halting cell growth rather than causing cell death, sirolimus may promote a more favorable vascular healing response compared to paclitaxel, potentially reducing inflammation and supporting earlier endothelial recovery. This mechanism could be particularly beneficial in the small-diameter, slow-flow environment of infrapopliteal arteries, where maintaining vessel patency and minimizing intimal hyperplasia are critical for long-term treatment success. The first-in-human experience using RST before paclitaxel DCB angioplasty in infrapopliteal arteries demonstrated favorable efficacy and safety results [24]. No early mortality or major amputations were reported, and the treated vessels maintained an 89% patency rate at the 1-year follow-up. Thus, the combination of RST with sirolimus delivery, as employed in this study, appears to provide results comparable to RST with paclitaxel delivery, without the potential risks associated with paclitaxel or permanent implants.

## Limitations

Despite these promising findings, some limitations of this study must be acknowledged. First, the single-arm, single-center design with a relatively small sample size may limit the generalizability of our findings, which would ideally be confirmed in a larger multicenter trial. Second, vessel recoil was measured in a subset of patients, and thus the robustness of these findings may also be limited. Third, the use of multiple imaging modalities including intravascular ultrasound, optical coherence tomography, and angiography for vessel measurements and device sizing may have introduced variability in the assessment of eligibility criteria. Fourth, this study evaluated RST combined with a sirolimus-based DCB. Thus, the effects of RST and sirolimus-based DCB cannot be independently evaluated with this study design. Finally, lack of a control group precludes direct comparison to standard therapies, making it difficult to definitively determine the effectiveness of this approach.

## Conclusions

The DEEPER LIMUS pilot study demonstrated promising safety and performance of RST combined with a sirolimus-based DCB for treating infrapopliteal lesions. This novel approach was associated with negligible acute vessel recoil and high rates of limb salvage, vessel patency, and wound healing through 1 year. While these data require confirmation in larger trials, this management strategy represents a promising addition to the endovascular armamentarium for treating infrapopliteal disease.
